# Personality and the use of cancer screenings. A systematic review

**DOI:** 10.1371/journal.pone.0244655

**Published:** 2020-12-28

**Authors:** André Hajek, Benedikt Kretzler, Hans-Helmut König

**Affiliations:** Department of Health Economics and Health Services Research, University Medical Center Hamburg-Eppendorf, Hamburg, Germany; University of Edinburgh, UNITED KINGDOM

## Abstract

**Background:**

No systematic review exists synthesizing studies examining the association between personality factors and use of cancer screenings. Hence, the aim of this systematic review is to provide an overview of empirical findings from observational studies investigating the link between personality factors (in terms of agreeableness, conscientiousness, extraversion, neuroticism and openness to experience) and use of cancer screenings.

**Methods:**

Medline, PsycInfo and CINAHL were searched using predefined search terms. Observational studies examining the link between personality factors and use of cancer screenings using validated tools were included. Study selection, data extraction, and quality assessment were performed by two reviewers.

**Results:**

In total, n = 11 studies were included in our systematic review. There is mostly inconclusive evidence regarding the link between agreeableness, neuroticism, openness to experience and the use of cancer screenings. Clearer evidence was identified for an association between increased extraversion and an increased use of cancer screenings. Moreover, the majority of studies identified a link between increased conscientiousness and an increased use of cancer screenings.

**Discussion:**

Studies indicate that personality factors, particularly an increased extraversion and increased conscientiousness, are associated with an increased use of cancer screenings. This knowledge may be beneficial to address individuals at risk for underuse.

**PROSPERO registration number:**

CRD42020176830

## 1. Introduction

Globally, cancer is a leading cause of death [1]. In light of the demographic ageing and the association between increased age and several cancer types, it is expected that the prevalence of cancer will increase [2]. On the other side, survival rates increase–particularly due to advancements in therapy and prevention efforts [3].

According to the World Health Organization [4], “30–50% of all cancer cases are preventable” (e.g., by adopting healthy lifestyles). Against this backdrop, it is important to note that primary prevention refers to the prevention of the occurrence of a disease such as cancer. Moreover, the goal of secondary preventions is the early detection and treatment of diseases such as cancer. This is important since early diagnosis of cancer can markedly increase the likelihood of successful treatment. Early signs of cancer include, amongst others, lumps, abnormal bleeding or sores that fail to heal. With regard to cancer, secondary prevention strategies include, amongst others, colorectal cancer screening or PSA (Prostate-specific antigen) test. It should be noted that early diagnosis is particularly relevant for cancers of the cervix, larynx, colon and rectum, breast and skin.

Given the fact that more than 14 million people are diagnosed with cancer every year and nearly 9 million individuals died from cancer in 2015, the World Health Organization has developed criteria and guidelines for screening [5]. A recent study [6] summarized the recommendations for cancer screening among 21 high income countries. They found that guidelines for cancer screening somewhat differ between these countries. While there were similar recommendations for well-established screening programs (like cervical and breast), greater variation between the countries were present regarding colorectal, prostate, lung and skin cancer screening. Further details are provided by Ebell et al. [6].

When their efficacy has been shown, screenings are usually paid for by health insurances in various countries. Furthermore, governments of numerous countries promote the use of several cancer screenings. Nonetheless, it has been shown that cancer screening rates are rather low in Germany [7]. Rather low (breast cancer) screening rates have been identified in various other European countries [8]. However, it should be noted that these rates could vary depending on the country and the type of cancer [8]. Various sociodemographic [9, 10] and need factors (e.g., morbidity or self-rated health) are associated with the use of cancer screenings [11, 12]. One widely used theoretical framework is the Andersen model for healthcare utilization [13]. This model distinguishes between predisposing characteristics (e.g., age), enabling resources (e.g., income or access to healthcare services) and need factors such as self-rated health. Some other studies used the health belief model as theoretical background to study the association between several psychological variables like perceived efficacy, perceived vulnerability or risk and the use of cancer screenings [14–18].

Thus far, several studies have shown an association between personality characteristics (i.e., big five personality traits: agreeableness, conscientiousness, extraversion, neuroticism and openness to experience [19]) and the use of cancer screenings [20, 21]. While it should be acknowledged that additional models are present [22], most commonly the personality factors are divided into the aforementioned big five traits [23]. Similarly, previous studies have also shown a link between personality and general health screenings (e.g., [24]) or general health care use (e.g., [25, 26]).

In short, conscientious individuals tend to be task- and goal-directed, follow the rules and are planful. Neuroticism is commonly associated with anger, depression or anxiety. Individuals scoring high in openness tend to be more prone to be complex, open for new ideas and tend to be creative. Extraverted individuals tend to be active and sociable. Lastly, agreeable individuals tend to be warm and altruistic. It is worth noting that neuroticism is sometimes called emotional instability or inverse emotional stability and openness to experience is sometimes called intellect or intellect/imagination.

There is no systematic review analyzing the association between personality factors and the use of cancer screenings. Therefore, our aim was to provide an overview of findings from observational studies (cross-sectional and longitudinal) investigating this link (covering screening procedures for the early detection of any cancer types). This knowledge may be beneficial to address individuals at risk for underuse. In this study, we focus on the well-known and widely acknowledged big five personality factors.

With regard to literature regarding the link between personality (in terms of the big five personality traits) and general health care use, previous research showed a link between personality factors (particularly neuroticism) and health care use [25, 27]. First, the positive association between agreeableness and increased use of alternative or complementary medicine [28] may be explained by the fact that individuals with high levels of agreeableness may tend to avoid conflicts with physicians and may therefore follow the recommendations provided by the physician [27]. The association between conscientiousness and health care use may be explained by health-promotion behavior [29] and a low rate of accidents [30] associated with increased levels of conscientiousness. The positive association between extraversion and health care use (e.g., hospitalization [26]) may be explained by the injury-prone behavior [31] and bad lifestyle habits [32] associated with high levels of extraversion. The positive association between neuroticism and health care use [26] can be explained by negative feelings which in turn could drive health care use [27]. Lastly, it has been shown that openness to experience is particularly associated with increased use of alternative or complementary medicine [33]. This may be explained by the fact that high levels of openness to experience reflect that these individuals tend to be open to various experiences such as traveling [31]. It may also be the case that these individuals are open to alternative or complementary medicine [27]. With regard to the specific link between personality and cancer screenings, further details are provided in the discussion section.

## 2. Materials and methods

This systematic review was conducted in line with the Preferred Reporting Items for Systematic Reviews and Meta-Analysis Protocols guidelines [34]. The study is registered with the International Prospective Register of Systematic Reviews (PROSPERO, registration number: CRD42020176830).

### 2.1 Search strategy and selection criteria

In three databases (Medline, PsycINFO, CINAHL), a systematic literature search was performed in April 2020. The search query for Medline is given in Table 1.

**Table 1:** Search query (Medline).

#1	Personality [Title/Abstract]
#2	Big five[Title/Abstract]
#3	Extraversion
#4	Agreeableness
#5	Conscientiousness
#6	Neuroticism
#7	Emotional stability
#8	Openness
#9	#1 OR #2 OR #3 OR #4 OR #5 OR #6 OR #7 OR #8
#10	Preventive health care
#11	Preventive health service*
#12	#10 OR #11
#13	Cancer screening
#14	Melanoma screening
#15	Colonoscopy
#16	Pap
#17	Mammography
#18	FOBT
#19	Guaiac
#20	CRC screening
#21	Cervical screening
#22	Breast exam
#23	Flexible sigmoidoscopy
#24	PSA
#25	#13 OR #14 OR #15 OR #16 OR #17 OR #18 OR #19 OR #20 OR #21 OR #22 OR #23 OR #24
#26	#12 OR #25
#27	#9 AND #26

A two-step process ((1) title/abstract screening and (2) full-text screening) was used for evaluation of inclusion/exclusion. It was performed by two reviewers (AH, BK) using defined selection criteria. Moreover, the reference lists of the articles finally included in our systematic review were examined by two reviewers. In case of disagreement, discussion was used to achieve a consensus (or by including a third party (HHK)).

Inclusion criteria were as follows:

- observational studies (cross-sectional and longitudinal) examining the link between personality factors (one or more big five personality traits) and use of cancer screenings (irrespective of age)
- studies using validated tools to measure personality factors
- studies published in peer-reviewed journals (English or German language).

The exclusion criteria were as follows:

- studies not investigating the link between personality characteristics and use of cancer screenings
- studies exclusively investigating samples with a specific disorder (e.g., individuals with cancer)
- study design other than observational
- assessment of personality or use of cancer screening not appropriate (e.g., not using validated tools to assess personality factors like single item measures or unclear period of cancer screening)
- studies not based on big five personality measures
- studies published in language other than German or English
- studies not published in peer-reviewed journals

Restrictions were not applied regarding region or time of publication. Prior to final eligibility criteria, a pretest was performed (based on a sample of 100 titles/abstracts). However, eligibility criteria did not change.

### 2.2 Data extraction and analysis

The data extraction was conducted by one reviewer (BK). A second reviewer (AH) performed a cross-check. Consensus discussions were used to resolve disagreements (or by including a third party (HHK))—or by contacting the study authors.

Data extraction included study design, measurement of key variables, characteristics regarding the sample, statistical techniques and main findings with regard to the link between personality factors and use of cancer screenings. The key results are presented for each personality trait separately in the results section of this systematic review.

### 2.3 Quality assessment

To date, a consensus on a quality assessment tool for studies investigating the use of preventive health care services (or more broadly: health care utilization) does not exist. Consequently, we adapted recent checklists created by Stuhldreher et al. [35] and improved by Hohls et al. [36]. Two reviewers (AH, BK) conducted the quality assessment. In case of disagreement, discussions were used to achieve a consensus (or including a third party (HHK)).

## 3. Results

### 3.1 Overview: Included studies

**Fig 1:**
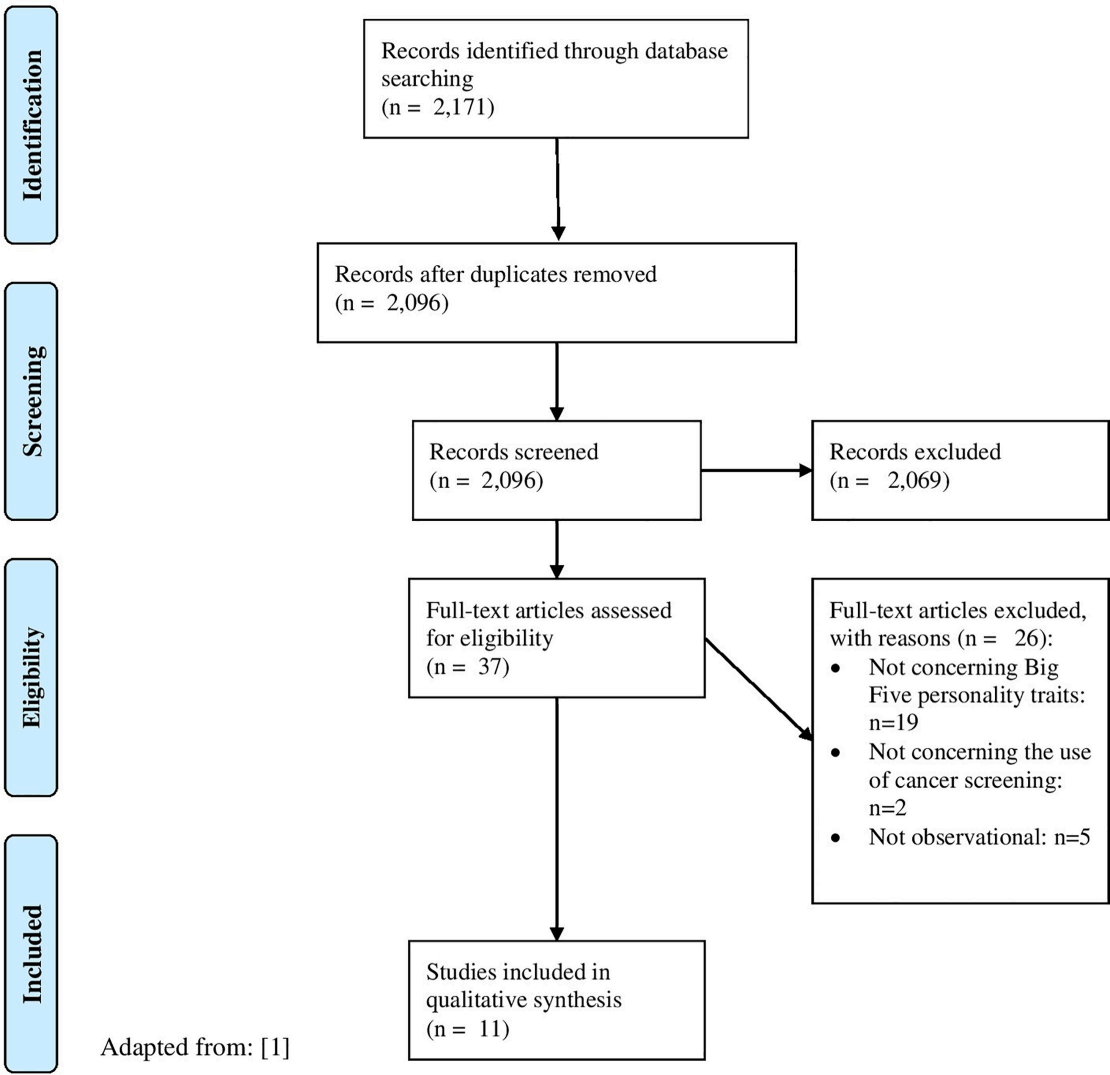
PRISMA flow diagram.

In Fig 1 (flow chart [37]), the study selection process is described. In sum, 37 full text articles were assessed for eligibility. It is worth noting that we also included studies to our full-text screening with unclear titles/abstracts. The main reasons for final exclusion were that they did not investigate the association between personality (in terms of the big five model) and cancer screenings. However, it is worth emphasizing that no studies were excluded because they used a different personality model (such as the HEXACO (Honesty-Humility, Emotionality, Extraversion, Agreeableness, Conscientiousness, and Openness to Experience) model [38]). Moreover, no studies were excluded because they used an invalid tool to assess personality or cancer screening. In total, n = 11 studies were included in our final synthesis (total number of observations: n = 338,091) [20, 21, 39–47]. The quality assessment of included studies is described in Table 2. A study overview and key findings are presented in Table 3. Findings of adjusted regression analyses (if possible) are also shown in Table 3. The key findings of our review are shown in Table 4.

**Table 2:** Quality assessment.

First author (year)	Study objective	Inclusion and exclusion criteria	Cancer screening description	Comparison group or disorder-specific HCU	Data source	Missing data	Statistics	Consideration of confounders	Sensitivity analysis	Sample size (subgroup)	Demographics	Results discussed with respect to other studies	Results discussed regarding generalizability	Limitations	Conclusion supported by data	Conflict of interest / funders	% of criteria fulfilled by study
Arai (2009) [39]	✓	✓	✓	✓	✓	✓	✓	✓	✓	✓	✓	✓	✓	✓	✓	✓	100
Aschwanden (2019) [20]	✓	✓	✓	✓	✓	X	✓	✓	✓	✓	✓	✓	✓	✓	✓	✓	93.8
Costa (2018) [40]	✓	✓	✓	✓	✓	X	✓	✓	X	✓	✓	✓	X	✓	✓	✓	81.3
Gale (2015) [41]	✓	✓	✓	✓	✓	X	✓	✓	✓	✓	✓	✓	✓	✓	✓	✓	93.8
Hill (2011) [42]	✓	✓	✓	✓	✓	X	✓	✓	X	✓	✓	✓	✓	✓	✓	✓	87.5
Hill (2013) [43]	✓	✓	✓	✓	✓	X	✓	✓	✓	✓	✓	✓	✓	✓	✓	✓	93.8
Niedzwiedz (2020) [44]	✓	✓	✓	✓	✓	✓	✓	✓	✓	✓	✓	✓	✓	✓	✓	✓	100.0
Nolan (2019) [21]	✓	✓	✓	✓	✓	X	✓	✓	✓	✓	✓	✓	✓	✓	✓	✓	93.8
Pandhi (2016) [45]	✓	✓	✓	✓	✓	X	✓	✓	X	✓	✓	✓	✓	✓	✓	✓	87.5
Schwartz (1999) [46]	✓	✓	✓	✓	✓	X	✓	✓	X	✓	✓	✓	✓	✓	✓	✓	87.5
Sen (2016) [47]	✓	✓	✓	✓	✓	X	✓	✓	✓	✓	✓	✓	✓	✓	✓	✓	93.8
% of criteria fulfilled by studies	100.0	100.0	100.0	100.0	100.0	18.2	100.0	100.0	63.6	100.0	100.0	100.0	90.9	100.0	100.0	100.0	

Notes: x = not fulfilled; ✓ = fulfilled

**Table 3:** Study overview and main findings.

First author	Country	Assessment of personality	Assessment of cancer screening utilization	Study type	Sample description	Sample size	Age	Females in total sample	Main findings
Arai (2009) [39]	Japan	Eysenck Personality Questionnaire-Revised (48 items)	gastric cancer screening (last five years)	cross-sectional	Miyagi Cohort study	n = 21,911	40–64 M = 51 SD not specified	55%	Logistic regression showed that the quartile with the highest extraversion score had higher odds of gastric cancer screening attendance (OR = 1.16, 95% CI: 1.07–1.26).
Aschwanden (2019) [20]	United States	Midlife Development Inventory (31 items)	breast, cervical, prostate and colorectal cancer screening (last two years, expect colorectal screening which was assessed for four years)	cross-sectional	Health and Retirement Study	n = 14,394	50–102 M = 68.1 SD = 10.6	58.6%	Controlling for demographic and socioeconomic covariates, logistic regression revealed that increased conscientiousness was related with increased odds of breast (OR = 1.13, 95% CI: 1.07–1.20), cervical (OR = 1.14, 95% CI: 1.07–1.20) and prostate cancer screening (OR = 1.08, 95% CI: 1.01–1.16). Higher neuroticism was associated with colorectal screening (OR = 1.05, 95% CI: 1.01–1.09). Increased extraversion was related with higher odds of breast (OR = 1.14, 95% CI: 1.16–1.22), cervical (OR = 1.17, 95% CI: 1.10–1.25) and colorectal screening (OR = 1.06, 95% CI: 1.01–1.12).
Costa (2018) [40]	Italy	Big Five Inventory (44 items)	screening barriers questionnaire (25 items) for Pap test barriers and self-sampling barriers	cross-sectional	people who attended Pap test screening at a regular hospital	n = 206	18–60 M = 37.9 SD = 13.2	100.0%	Regression analysis explored that higher conscientiousness was significantly associated with lower Pap test screening barriers (ß = -0.18, p<0.05).
Gale (2015) [41]	United Kingdom	Midlife Development Inventory, version of the Health and Retirement Study (26 items)	use of a home bowel cancer testing kit (period not specified)	cross-sectional	English Longitudinal Study of Ageing	n = 2,681	60–75 for participants in bowel screening (n = 1,539): M = 65.8 SD = 3.9 for non-participants (n = 1,142): M = 67.7 SD = 5.1	for participants in bowel screening (n = 1,539): 57.4% for non-participants (n = 1,142): 52.0%	In the fully-adjusted regression model, no personality trait was significantly associated with participation in bowel cancer screening.
Hill (2011) [42]	Canada	Big Five Inventory (44 items)	screening barriers questionnaire (25 items) for Pap test barriers and self-sampling barriers	cross-sectional	undergraduate students	n = 257	17–45 M = 20.3 SD = 3.8	100.0%	Hierarchical multiple linear regression revealed that increased extraversion (ß = -0.12, p<0.05) and increased conscientiousness (ß = -0.17, p<0.01) were inversely related to pap test barriers.
Hill (2013) [43]	Canada	neuroticism subscale of the Big Five Inventory (eight items)	Pap test participation (period not specified)	cross-sectional	undergraduate students	n = 257	17–45 M = 20.3 SD = 3.8	100.0%	Hierarchical regression explored that neuroticism was not significantly associated with Pap test participation.
Niedzwiedz (2020) [44]	United Kingdom	Eysenck Personality Inventory Neuroticism Scale (twelve items)	mammogram or cervical smear test (lifetime)	longitudinal	UK Biobank	breast cancer screening: n = 143,461 cervical screening: n = 141,753	breast cancer screening: 50–70 age groups: 50–54: 30,942 (21.6%) 55–59: 35,614 (24.8%) 60–64: 45,485 (31.7) 65+: 31,420 (21.9%) cervical screening: age groups: <45: 19,391 (13.7%) 45–49: 25,431 (17.9%) 50–54: 28,753 (20.3%) 55–59: 30,991 (21.9%) 60–64: 37,187 (26.2%)	100.0%	Logistic regression revealed that increased neuroticism was significantly associated with breast cancer screening (OR = 1.01, 95% CI: 1.00–1.02) and cervical screening (OR = 0.99, 95% CI: 0.98–0.99).
Nolan (2019) [21]	Ireland	NEO-Five Factor Inventory (60 items)	breast lump check, mammogram, prostate examination and PSA blood test (lifetime)	cross- sectional	Irish Longitudinal Study on Ageing	5,522	50–93 M = 63.6 SD = 10.0	51.8%	For the age group from 50 to 64, regression analysis showed that increased extraversion was associated with using breast lump check (IRR = 1.06, p<0.01) and PSA blood test (IRR = 1.03, p<0.1). PSA blood test was related to higher openness (IRR = 1.00, p<0.05) and lower agreeableness (IRR = 0.99, p<0.01). Higher conscientiousness was associated with prostate examination (OR = 1.04, p<0.01). For people older than 65, regression analysis revealed that higher openness was related to breast lump check (OR = 1.12, p<0.01) and mammogram (OR = 1.08, p<0.01). Increased conscientiousness was associated with prostate examination (OR = 1.07, p<0.1).
Pandhi (2016) [45]	United States of America	Big Five Inventory (29 items)	mammogram (last twelve months)	cross- sectional	Wisconsin Longitudinal Study	n = 6,975	age groups: <60: n = 583 (8%) 60–64: n = 3,674 (53%) 65–69: n = 2,149 (31%) ≥70: n = 569 (8%)	54%	People with lower conscientiousness had a significantly lower probability of mammogram attendance (p = 80%, 95% CI: 77%-83%), and people with higher conscientiousness had a significantly higher probability (85%, 95% CI: 82%-87%). Decreased agreeableness was related to decreased probability of mammogram attendance (77%, 95% CI: 73%-81%). Increased agreeableness was associated with higher chances of taking mammography (84%, 95% CI: 82%-87%).
Schwartz (1999) [46]	United States of America	conscientiousness subscale of the Neuroticism, Extroversion, and Openness Five Factor Inventory (twelve items)	mammogram (last twelve months)	cross- sectional	women with a family history of breast cancer	n = 200	40–84 M = 57	100.0%	Hierarchical logistic regression revealed that increased conscientiousness was marginally significantly associated with mammogram utilization (OR = 0.74, 95% CI: 0.21–2.56).
Sen (2016) [47]	United States of America	conscientiousness (five items) and neuroticism (one item)	mammogram attendance (last two years)	longitudinal	National Survey of Midlife Development in the United States	n = 474	range not specified M = 57.3 SD = 10.5	100.0%	Logistic regression showed that increased conscientiousness was associated with mammogram attendance (OR = 2.13, 95% CI: 1.23–3.69).

**Table 4:** Key findings.

Personality traits	Number of studies	Positive relationship	Negative relationship	No relationship
Agreeableness	6	1	0	5
Conscientiousness	8	5	1	2
Extraversion	7	3	0	4
Neuroticism	10	1	0	9
Openness to experience	6	0	0	6

Studies (and data) came from North America (n = 6, with: Canada, n = 2; United States, n = 4) and Europe (n = 4; two studies from the United Kingdom, one study from Italy, and one study from Ireland) and Asia (n = 1, Japan). Nine cross-sectional and two longitudinal studies have been included. Different instruments were used to quantify personality characteristics (e.g., Big Five Inventory with 29 items or the Eysenck Personality Questionnaire-Revised with 48 items) [45]. While Schwartz [46] only focused on conscientiousness (in the association with use of mammography), Sen [47] concentrated on conscientiousness and neuroticism (in the association with mammogram attendance), and Hill [43] only focused on neuroticism (in the association with the use of Pap test), the remaining eight studies included all five personality factors. Some studies focused on specific cancer screenings such as Pap test [40, 42, 43], mammography [45–47], gastric cancer screening [39] or bowel cancer screening [41], whereas other studies more generally investigated the link between personality factors and use of different cancer screening procedures [20, 21, 44]. Two of the eleven studies focused on screening barriers [40, 42], both using a questionnaire (25 items) for Pap test barriers and self-sampling barriers (for example including questions such as “This type of screening is too time-consuming” or “This type of screening would be embarrassing for me”). Since these studies focused on barriers rather than actual use, we will describe them separately in the next subsections of the results. Moreover, we qualitatively examined whether the findings presented in the sections 3.3 to 3.7 differ by personality measures, screenings, sample sizes or sample age. However, we did not identify any systematic differences. Nevertheless, due to the small number of studies, these preliminary findings should be interpreted with great caution. If data permit, future meta-analyses (including meta-regressions) are required to verify our assumptions.

The age ranged from 17 to 102 years across the studies. However, most of the studies focused on individuals aged 50 and over. Only the two studies from Hill focused on undergraduate students. While some studies focused on mammography or Pap test (as stated above) and therefore exclusively examined women (100%) [40, 42–44, 46, 47], the proportion of women was slightly higher than 50% in most of the remaining included studies. The sample size ranged from 200 to 21,911. Additional details are presented in Table 3.

### 3.2 Quality assessment

The studies included in our review fulfilled between 81% and 100% of the criteria. The categories with the most unfulfilled criteria were ‘handling of missing data’ (18.2%) and ‘performed sensitivity analysis’ (64%). Please see Table 2 for further details.

In the next sections, we display our key results separately for each personality factor: (1) agreeableness, (2) conscientiousness, (3) extraversion, (4), neuroticism and (5) openness to experience. If possible, two decimal places are reported in Table 3 and in the following sections. However, some studies only reported one decimal place. In these cases, only one decimal place was reported.

### 3.3 Agreeableness and use of cancer screenings

In total, n = 6 studies investigated the link between agreeableness and use of different cancer screenings. Two out of these six studies found an association between agreeableness and cancer screenings: One study [21] showed that PSA blood test was associated with lower agreeableness (IRR = 0.99, p<0.01) among individuals from 50 to 64 years. Another study [45] found an association between a higher probability of having a mammogram and increased agreeableness.

Furthermore, the n = 2 studies [40, 42] focusing on screening barriers did not find a link between agreeableness and screening barriers.

### 3.4 Conscientiousness and use of cancer screenings

In total, n = 8 studies examined the association between conscientiousness and use of different cancer screenings. Five out of these eight studies found an association between increased conscientiousness and use of different cancer screenings (particularly for mammogram utilization). For example, Schwartz [46] showed that increased conscientiousness was marginally significantly associated with mammogram utilization (OR = 0.74, 95% CI: 0.21–2.56). Another study [47] showed that increased conscientiousness was associated with mammogram attendance (OR = 2.13, 95% CI: 1.23–3.69). Similar findings were made by Pandhi et al. [45]. Aschwanden et al. [20] also showed an association between increased conscientiousness and increased odds of breast (OR = 1.13, 95% CI: 1.07–1.20), cervical (OR = 1.14, 95% CI: 1.07–1.20) and prostate cancer screening (OR = 1.08, 95% CI: 1.01–1.16). In the same vein, Nolan et al. [21] found an association between higher conscientiousness and use of prostate examination among individuals from 50 to 64 years (IRR = 1.04, p<0.01) and individuals aged 65 years and over (IRR = 1.07, p<0.1).

The n = 2 studies [40, 42] focusing on screening barriers both found an association between higher conscientiousness and lower Pap test barriers (Costa et al. [40]: β = -0.18, p < .05; Hill et al. [42]: β = -0.17, p < .01).

### 3.5 Extraversion and use of cancer screenings

In total, n = 6 studies examined the association between extraversion and use of cancer screenings. Three out of these six studies found an association between higher extraversion and an increased likelihood of participation in cancer screenings. More precisely, Arai et al. [39] showed that the quartile with the highest extraversion score had higher odds of gastric cancer screening attendance (OR = 1.16, 95% CI: 1.07–1.26). Moreover, Aschwanden et al. [20] showed that increased extraversion was associated with higher odds of breast (OR = 1.14, 95% CI: 1.16–1.22), cervical (OR = 1.17, 95% CI: 1.10–1.25) and colorectal screening (OR = 1.06, 95% CI: 1.01–1.12). Furthermore, Nolan et al. [21] showed an association between increased extraversion and the likelihood of using breast lump check among individuals from 50 to 64 years (IRR = 1.06, p<0.01).

One [42] out of the n = 2 studies [40, 42] concentrating on screening barriers found that increased extraversion was associated with lower Pap test barriers (ß = -0.12, p<0.05).

### 3.6 Neuroticism and use of cancer screenings

In sum, n = 8 studies investigated the association between neuroticism and the use of cancer screenings. Two out of these eight studies showed an association between neuroticism and use of cancer screenings. One of these two studies showed a link between higher neuroticism and an increased likelihood of colorectal screening (OR = 1.05, 95% CI: 1.01–1.09) [20]. The second study [44] showed an association between increased neuroticism and an increased likelihood of breast cancer screening (OR = 1.01, 95% CI: 1.00–1.02), whereas increased neuroticism was associated with reduced cervical screening participation (OR = 0.99, 95% CI: 0.98–0.99) [44].

An association between neuroticism and Pap test barriers was not present in the n = 2 studies [40, 42] which examined screening barriers.

### 3.7 Openness to experience and use of cancer screenings

In sum, n = 6 studies examined the association between openness to experience and use of cancer screenings. Only one out of these six studies found an association between openness to experience and use of cancer screenings. The study performed by Nolan et al. [21] found that higher openness was associated with the use of PSA blood test (IRR: 1.002, p < .05) among individuals 50 to 64 years. Furthermore, they found an association between higher openness and use of breast lump check (IRR: 1.12, p < .01) and mammogram (IRR: 1.08, p < .01) [21].

The n = 2 studies [40, 42] which focused on screening barriers did not find an association between openness to experience and Pap test barriers.

## 4. Discussion

The purpose of this systematic review was to provide an overview of empirical findings from observational studies investigating the link between personality factors and use of cancer screenings. In total, only few studies exist which examined the association between personality factors and use of cancer screenings. More precisely, we included eleven studies in our systematic review [20, 21, 39–47].

In sum, there is mostly inconclusive evidence regarding the link between agreeableness, neuroticism, openness to experience and the use of cancer screenings. Previous studies mainly investigated the association between agreeableness and the use of cancer screening in an exploratory way (i.e., without prespecified hypotheses). However, the link between agreeableness and the use of cancer screenings (and more broadly, the use of preventive services such as flu vaccination) appears to be plausible because individuals who score high in agreeableness may tend to avoid conflicts with doctors in terms of decision-making [48]. Therefore, future research is needed to examine this association in far more detail. Future studies based on qualitative methods may also be important. These qualitative studies may reveal underlying reasons why individuals who score high in agreeableness may have a different cancer screening behavior.

An association between increased neuroticism and decreased use of cancer screenings appears to be plausible because of maladaptive behavior (avoidance). However, some individuals scoring high in neuroticism may have a higher likelihood of using cancer screenings since these individuals are driven by factors such as worry or anxiety about diseases. We think that future studies are required–for example, to test possible non-linear effects (e.g., curvilinear effects) of neuroticism on the use of cancer screenings. More precisely, it appears plausible that the greater the extreme of neuroticism (either low or high), the greater the likelihood of using cancer screenings. While the potential link between high neuroticism and cancer screenings has been described above, the potential link between low neuroticism (emotional stability) and cancer screenings may be driven by general self-esteem or general self-efficacy [49] which both are associated with a higher likelihood of health check-ups and cancer screenings [17, 50]. These curvilinear effects could be examined by using linear, quadratic and cubic terms for neuroticism in regression analysis.

Previous studies explained a link between openness to experience and the use of cancer screenings by the fact that openness to experience reflects variety, change and cognitive stimulation (e.g., [21]). Thus, individuals scoring high in openness to experience may be more open-minded when it comes to the use of cancer screenings. However, future studies are needed to examine this association in additional detail.

Clearer evidence was identified for an association between increased extraversion and an increased use of cancer screenings. Extraversion is associated with positive emotions. It may be the case that these individuals may have positive expectations regarding cancer screenings [20]. This appears plausible since it has been shown that positive emotions are associated with positive expectations [50–52]. Moreover, the link between positive expectation and increased use of cancer screenings may be explained by the goals individuals set in their daily lives [53]. However, future research is required to clarify the link between extraversion and cancer screening.

Moreover, the majority of the studies identified a link between increased conscientiousness and an increased use of cancer screenings (with strong evidence for mammography). This association appears very plausible because conscientious individuals tend to follow the rules and tend to be planful. Furthermore, they are task- and goal-directed. In the same vein, they may adhere to cancer screening recommendations.

In general, the quality of the studies included in our review was rather high. It should also be noted that most of the studies included adjusted for several important covariates (like socioeconomic or health-related covariates). Main restrictions were that some studies did not perform robustness checks. Nevertheless, we recommend that future studies should check the robustness of their findings–which is also in accordance with current guidelines [54]. Furthermore, only two studies described how they handled missing data in their analyses [39, 44]. Upcoming research should clarify how they treated missing data. Ideally, future studies should overcome these limitations by using techniques such as full-information maximum likelihood or multiple imputation [55, 56].

A few factors somewhat restrict the comparability of the studies finally included in our systematic review. There was some variety in tools used to quantify personality characteristics. For instance, Nolan et al. [21] used the NEO-FFI (60-item version) [57], whereas Gale et al. [41] used the Midlife Development Inventory (version of the Health and Retirement Study) with 26 items [58]. However, since all of these tools rely on the five factor model and are well validated (in accordance to our inclusion criteria), this should not restrict the comparability of our results.

There was also some variety in the outcome measures (cancer screenings). For example, while some studies (e.g., [46, 47]) exclusively focused on the use of mammography, other studies more broadly examined the use of cancer screenings such as breast, cervical, prostate and colorectal cancer screening (e.g., [20]). The recall period for cancer screening ranged from twelve months [45, 46] to lifetime [21, 44]. Two studies used the “screening barriers questionnaire” (25 items) developed by Hill and Gick [42]. Cronbach’s alpha for Pap test barriers was .89 and Cronbach’s alpha for self-sampling barriers was .84 [42].

Future research is necessary to examine the link between personality factors and all sorts of cancer screening in further detail since the strength of the association between factors like conscientiousness and different sorts of cancer screenings may vary. The bulk of the evidence stems from cross-sectional studies, whereas only two longitudinal studies [44, 47] have been identified. Therefore, we strongly recommend future studies based on longitudinal data to confirm the existing findings. Furthermore, data exclusively came from North America, Europe and Asia (Japan). Thus, it remains an open question whether personality factors are associated with the use of cancer screenings, for example, in South America or Africa. Beyond cultural differences, the health care system may also have an impact on the link between personality factors and cancer screenings.

Some strengths and limitations of our systematic review are worth emphasizing. This is the first systematic review synthesizing the findings from observational studies with regard to personality and the use of cancer screenings (including screening procedures for the early detection of any cancer type). We performed a quality assessment. Moreover, we focused on observational data and excluded illness-specific samples which can increase the generalizability. Study selection, data extraction and quality assessment were performed by two reviewers. A meta-analysis could not be performed due to study heterogeneity (e.g., in the outcomes used in the studies). This is in accordance with the recommendations given by Egger and colleagues [59]. They recommended great caution when conducting a meta-analysis, particularly based on observational studies because they can result in incorrect estimations for several reasons [59]. Nevertheless, it should be acknowledged that recommendations differ for meta-analysis based on observational studies [60]. Furthermore, we concentrated on the well-known and widely acknowledged big five personality factors. Nevertheless, other studies, for example Hajek et al., showed a link between, for example, self-esteem and the use of cancer screenings [61]. Therefore, future studies are required to clarify the association between other psychological factors and the use of cancer screenings. We included several cancer screenings in our search strategy and additionally performed a hand search. However, it should be noted that the search strategy may be somewhat restricted (e.g., “intellect/imagination” was not used as a synonym for “openness”). Moreover, neither MeSH headings (Medline and CINAHL) nor the thesaurus function (PsycINFO) was used.

## Conclusions

Studies included in our systematic review indicate that personality factors, particularly an increased extraversion and—most strikingly—increased conscientiousness, are associated with an increased use of cancer screenings. This knowledge may be beneficial to address individuals at risk for underuse. More precisely, one may conclude that health care providers such as physicians (particularly including general practitioners or geriatricians), nurses, psychologists, or staff in nursing homes should routinely assess personality traits of individuals. This could assist in identifying individuals at risk for underuse.

Thus far, only few, mainly cross-sectional, studies examined the link between personality factors and the use of different cancer screenings. Given the fact that personality factors can change over time, future research based on longitudinal data is urgently required to gain further insights into the link between personality factors and the use of cancer screenings. Moreover, based on the health belief model [62], potentially important factors (e.g., self-efficacy) could be examined in the link between personality and prevention behavior [63] such as cancer screenings.

Empirical studies included in our review stemmed from high-income countries such as the United States, Canada, the United Kingdom or Japan. Future research in low income or developing countries is urgently required (where certain restrictions may exist such as poor access to healthcare or financial barriers).

## Supporting information

S1 Checklist PRISMA 2009 checklist. (DOC)
